# CALENDAR

**DOI:** 10.1054/bjoc.1999.1050

**Published:** 2000-02-01


					26 March - 1 April 2000
European School of Genetic Medicine Scientific
Programme of the 13th Course in Medical Genetics
Genova, Italy
Course programme:
26 March 2000 Introduction to Genetic Analysis
27 March 2000 Genetics of Complex Traits
28 March 2000 Molecular Genetics
29 March 2000 Clinical Genetics
30 March 2000 Neurogenetics
31 March 2000 Genome Projects
1 April 2000 Genetic Medicine in Year 2000
Further information from:
Alessandro Palmesino, European Genetics Foundation, Palazzo
del Melograno, Piazza Campetto 2/8, 16123 Genova, Italy.
Tel: + 39 010 246 46 46, + 39 010 246 41 66; Fax: + 39 010 246
60 55; E-mail: eurgef@tin.iti; Website: http://www.eurogene.org
5-9 April 2000
Eurogin 2000
Global Challenge of Cervical Cancer Prevention
4th International Multidisciplinary Congress
Paris, France
Further information from:
Congress Secretariat, BAXON, N. Duraincie, C Boussin,
220-224 boulevard Jean, Jaures, 92773 Boulogne Cedex, France.
Tel: + 33 (0) 15520 23 83; Fax: + 33 (0) 1552023 93;
E-mail: duraincie.baxon@covos.fr
27 September - 1 October 2000
Pigment Cell research - Perspectives for the Third
Millenium
University of Ulm/Donau, Germany
Further information from:
Department of Dermatology, University of Ulm, ESPCR 2000,
89070 Ulm/Donau, Germany. Tel: + 49 (0) 731 50 22251;
Fax: + 49 (0) 731 50 23772; E-mail: ralf.peter@medizin.uni-
ulm.dei; Website: http://www.uni-ulm.de/klinik/dermatologie
10-13 July 2000
29th British Congress of Obstetrics and Gynaecology
International Convention Centre
Birmingham, UK
Further information:
BCOG Secretariat, Congress House, 65 West Drive, Cheam,
Sutton, Surrey SM2 7NB, UK. Tel: + 44 (0) 20 8661 0877;
Fax: + 44 (0) 20 8661 9036; E-mail: info@conforg.com
Errata
Br J Cancer 80(11): 1689-1696
Fractionated -irradiation renders tumour cells more responsive to
apoptotic signals through CD95
MA Sheard, PH Krammer and J Zaloudik
Figure 2A was reconfigured during the editorial process from
vertical columns to horizontal rows, inadvertently rendering the
text inconsistent with the layout of the Figure. References in the
text to the `top panels' of Figure 2A should be read as `left
panels', and `bottom panels' should be read as `right panels'.
Br J Cancer 81(5): 835-840
Chemotherapy for unresectable and recurrent intramedullary glial
tumours in children
V Doireau, J Grill, M Zerah, A Lellouch-Tubiana, D Couanet,
P Chastagner, JC Marchal, Y Grignon, Z Chouffai and C Kalifa
for the Brain Tumours Subcommittee of the French Society of
Paediatric Oncology (SFOP)
There was an error in the conversion of the doses of procarbazine
from mg kg-1 to mg m-2. The dose in mg kg-1 is correct, i.e. 4
mg kg-1 but the dose in mg m-2 should be 120 mg m-2 and not
200 mg m-2 as mentioned in the paper. To summarize, the doses
of chemotherapy to be used in this protocol are:
CARBOPLATIN 15 mg kg-1 or 450 mg m-2 on
DAY 1
PROCARBAZINE 4 mg kg-1 or 120 mg m-2 on
DAY 1 TO DAY 7
ETOPOSIDE 5 mg kg-1 or 150 mg m-2 on
DAY 22 AND DAY 23
CISPLATIN 1 mg kg-1 or 30 mg m-2 on
DAY 22 AND DAY 23
VINCRISTINE 0.05 mg kg-1 or 1.5 mg m-2 on
DAY 43
CYCLOPHOSPHAMIDE 50 mg kg-1 or 1500 mg m-2 on
DAY 43.
For further information concerning the modalities of drug admin-
istration, the readers are invited to contact the authors at the
address indicated in the paper.
CALENDAR
1122
British Journal of Cancer (2000) 82(5), 1122
(c) 2000 Cancer Research Campaign
DOI: 10.1054/ bjoc.1999.1050, available online at http://www.idealibrary.com on

				

